# Intracortical Administration of the Complement C3 Receptor Antagonist Trifluoroacetate Modulates Microglia Reaction after Brain Injury

**DOI:** 10.1155/2019/1071036

**Published:** 2019-11-14

**Authors:** Roxana Surugiu, Bogdan Catalin, Danut Dumbrava, Andrei Gresita, Denisa Greta Olaru, Dirk M. Hermann, Aurel Popa-Wagner

**Affiliations:** ^1^Center of Clinical and Experimental Medicine, University of Medicine and Pharmacy of Craiova, Craiova, Romania; ^2^Experimental Research Centre for Normal and Pathological Aging, University of Medicine and Pharmacy of Craiova, Romania; ^3^Department of Anatomy, University of Medicine and Pharmacy of Craiova, Craiova, Romania; ^4^Chair of Vascular Neurology, Dementia and Ageing Research, Department of Neurology, University of Duisburg-Essen, University Hospital Essen, Germany

## Abstract

Worldwide, millions of individuals suffer an ischemic stroke each year, causing major disability, especially in the elderly, where stroke is the number one cause of disability. However, to date, no effective therapy exists that targets the functional recovery after stroke. After necrosis, neuroinflammation is a common feature of the acute stroke and a major obstacle to tissue restoration. In the lesioned area, the dying neurons release chemotactic signals, such as fractalkine/CX3CL1, which evoke “eat-me” signals that are recognized by microglia expressing complement C3a receptor (C3aR), resulting in phagocytosis of the dying but still viable neurons, known as secondary phagocytosis. Using a mouse model of stroke and two-photon microscopy, we aimed to attenuate poststroke phagocytosis of the dying but still viable neurons by using SB 290157, an antagonist of C3aR. We found that intracortical administration of SB 290157 reduced the number of inflammatory microglial cells expressing ED1 and Iba1 antigens at the lesion site. We could show, in vivo, that two days after a needle-induced cortical lesion there were less microglial cells present around the injury site, displaying less high-order branches and an increase in the lower order ones, suggesting an attenuated phagocytic phenotype in treated animals as compared with controls. We conclude that the C3aR antagonist, SB 290157, may be used in the future to limit the neuronal death by limiting secondary phagocytosis after stroke.

## 1. Introduction

Worldwide, millions of individuals suffer an ischemic stroke each year, causing major disabilities, especially in the elderly [1, 2]. Although several therapeutic approaches such as artery reperfusion and carotid endarterectomy have good results during the acute phase, the majority of stroke patients do not benefit from them [3]. Moreover, to date, no effective therapy exists that targets the long-term functional recovery after stroke [2, 4].

After necrosis, neuroinflammation is a common feature of acute stroke and a major obstacle to brain tissue restoration [5]. The long-lasting and multiphasic inflammatory reaction after stroke involves the recruitment of local microglia. Under pathological conditions, microglial functions are largely dependent on activation stimuli. However, the inhibition of microglial activation and proliferation is also undesirable as short-lived mild microglial activation might help in tissue preservation, repair, and renewal [6, 7]. As such, there is an increased need for therapies aimed specific at promoting cellular survival in the penumbra by modulating poststroke inflammation [5, 8].

Neuroinflammation is an extremely complex process, which we do not fully understand. For example, in normal conditions, microglial cells play an important role in the regulation of homeostasis in physiological conditions which, due to their highly dynamic properties, constantly check the surface of neurons in order to detect microlesions at this level [9–11]. In pathological conditions, the dying neurons release chemotactic signals, such as fractalkine/CX3CL1, which evoke “eat-me” signals that are recognized by microglia, resulting in phagocytosis of the dying neurons, known as secondary phagocytosis [12, 13]. However, the central nervous system inflammatory response to brain injury is not only mediated by tissue-specific pathways but also by systemic ones, such as the immune classical pathway. Postmortem investigation of brain tissue data showed an activation and accumulation of deposits of C1q, C3c, and C4d around ischemic lesions after stroke [14]. Furthermore, levels of serum complement proteins and activation products correlate with stroke severity [15, 16] and functional disability [17, 18]. Because C1q alone and/or in conjunction with C3 can promote microglial clearance of misfolded proteins and apoptotic neurons [19, 20] and potentially impact neurotoxic inflammatory gene expression [19], targeting the complement system could be a promising approach for immunomodulatory therapies.

There is a challenge to develop stroke therapies that modulate the capability of microglia conversion to phagocytes in the injured area by local administration of anti-inflammatory drugs [21]. In this study, we tested the efficacy of the C3aR antagonist, SB 290157, to limit the neuronal death by secondary phagocytosis after stroke following intracortical administration. Further, we examined the efficacy of SB 290157 to modulate microglia phenotype after a needle-induced cortical lesion.

## 2. Methods

### 2.1. Animals and Experimental Groups

Wild-type C57BL/6J mice were used to evaluate the minimum anti-inflammatory effect that different drug doses have on the central nervous system (CNS) after stroke. In total, 30 mice were used for this part of the study, divided into three groups: (a) one receiving half of the treatment dose (*n* = 10), (b) another receiving a third of the treatment dose (*n* = 10), and (c) a control one, which received only the vehicle (*n* = 10). To quantify the effect of the drug, in vivo, transgenic male CX_3_CR_1_^eGFP/-^ mice (Jung et al., 2000) were used. They were divided into four groups, receiving the full dose (*N* = 4), half (*N* = 4), a third (*N* = 4), and a sham (*N* = 5). All procedures and protocols described in the study were performed in the Animal Facility of the University of Medicine and Pharmacy from Craiova, in accordance with the guidelines of Romanian Experimental Animals Protection Law, and were approved by the Animal Experimentation Ethics Board of the University (152/26112015).

### 2.2. Treatment

After stroke induction, a stereotaxic instrument (Stoelting Co.) and a 1 *μ*l Hamilton syringe were used to inject 3x 1 *μ*l of a solution containing 1 mg trifluoroacetate/ml PBS/0.1% DMSO solution (SB 290157, trifluoroacetate salt—N2-[2-(2,2-diphenylethoxy) acetyl]-L-arginine trifluoroacetate salt). A second group received half of the full dose, and a third group received a third of the full dose. The control group went through the same procedures but received only vehicle. For the *in vivo* study using 2P microscopy, just one injection was used, with different doses, and microglia behaviour around the injected site was observed for 3 days. The antagonist potency (IC_50_) of SB 290157 at the mouse C3aR is 7 nM [22].

### 2.3. Permanent Middle Cerebral Artery Occlusion

Focal cerebral ischemia was induced by permanent occlusion of the right middle cerebral artery (MCA) as previously described [23]. Briefly, mice were anesthetized with 120 mg/kg Ketamine and 12 mg/kg Xylazine. After the skin and muscle were removed, a small craniotomy was performed over the MCA bifurcation, so that it can be easily accessed. MCA was permanently occluded using a thermocoagulator directly through the dura to avoid cortical damage caused by excessive heat.

### 2.4. Immunohistochemistry

Anesthetized mice were perfused intracardially with saline 5 ml saline, followed by 5 ml 4% paraformaldehyde and overnight fixation as this method has been proven to have the least impact on microglial activation [24]. After freezing procedures, 25 *μ*m thick sections were cut and prepared for immunohistochemistry following standard protocols, as previously described [25]. Antibodies against Iba-1 (1 : 3000, Wako Chemicals USA Inc., Richmond, VA, USA) were used to identify macrophages and microglia, 4-hydroxy-2-nonenal (4-HNE; Abcam, Cambridge, MA, USA), and anti-macrophage/monocyte antibody, clone ED-1 (1 : 1000, Sigma-Aldrich) to identify activated microglia. Negative controls that omit primary antibodies and positive controls were applied for each case. The positive cells were counted using a 40x magnification in serial coronal sections. Results are presented as Iba-1^+^ and ED-1^+^ cells per 100 *μ*m^2^ within areas measured from 40x images using Fiji software (National Institutes of Health) [26].

### 2.5. Determination of the Infarct Volume by Immunohistostaining

To assess the size of the infarct induced by focal ischemia, we used mouse anti-NeuN immunostaining. Every 20th free-floating section of 25 *μ*m was immunostained for neuronal nucleus marker NeuN, to cover the entire infarcted volume, which was then calculated as the sum of the partial areas using ImageJ. Briefly, the tissue was incubated with a mouse anti-NeuN (1 : 1000, Millipore, Germany) at 4°C overnight. On the next day, sections were rinsed with PBS and incubated with Alexa Fluor® 488 goat anti-mouse IgG. After a final rinsing, sections were brought to Superfrost Plus slides and mounted using PVA/DABCO-containing medium. Integration of the resulting partial volumes (partial areas × number of sections × section thickness × section intervals) yielded the total volume of the infarct as previously described [27].

### 2.6. 2-Photon Laser Scanning Microscopy

High -resolution, time-lapse *in vivo* imaging was performed using a 7MP Zeiss two-photon laser scanning microscope (2P-LSM) on anesthetized mice (120 mg/kg Ketamine and 12 mg/kg Xylazine). Prior to the imaging session, a cranial window was implanted over the right somatosensory cortex of the animal, using a previously described method [28]. Briefly, the skin and fat tissue was removed and a custom-made handler was fixed to the skull of the animals using dental cement. A small craniotomy was performed on the right parietal bone. After any bleeding was stopped, a twenty-minute 2P-LSM session was used as baseline. Before starting the imaging session, optimization was made by placing the animal on a special custom-made imaging table, capable of manual tilting the animal in *x* and *y* axis. 2P-LSM imaging was performed in 3 × 3 Z-stack planes of 415 × 415 *μ*m, down to a subdural depth of 75 to 150 *μ*m using a W-Plan Apochromat 20x/1.0 DIC Vis-IR M27 water immersion objective (×20, Carl Zeiss, Jena, Germany) controlled by ZEN 2010 imaging software (Carl Zeiss). The florescence was excited using an fs-pulsed titanium-sapphire laser (Chameleon Vision II, Coherent, Glasgow, UK) having a peak power higher than 3.5 W tuned to 910 nm [10]. A 500 × 500 × 1000 *μ*m injury was made using a small needle of a Hamilton syringe, and 1 *μ*l of SB 290157 (full, half, and third dose or vehicle) was slowly injected over a period of 60 seconds. After the injury was made, a round cover slip was applied and fixed with cyanoacrylate and dental cement for repetitive imaging. The same area was imaged for a period of twenty minutes, every 24 h, for 3 days. Further computerized analysis, such as microglia branch arborisation, was made using Fiji and its plugins [29] and Adobe InDesign (Adobe, USA).

### 2.7. Statistical Analysis

Statistical analysis was performed using GraphPad 6 and Microsoft Excel. Immunohistochemistry results were evaluated multiple comparison two-way ANOVA. For all morphological analyses, Mann-Whitney test was used. Unless noted otherwise, all figures show mean value and standard error of the mean (SEM) and the statistical significance is displayed as follows: ^∗^*p* < 0.05, ^∗∗^*p* < 0.01, and ^∗∗∗^*p* < 0.001.

## 3. Results

### 3.1. C3aR Antagonist Impacts Poststroke Inflammation but Not the Stroke Volume

**Figure 1:**
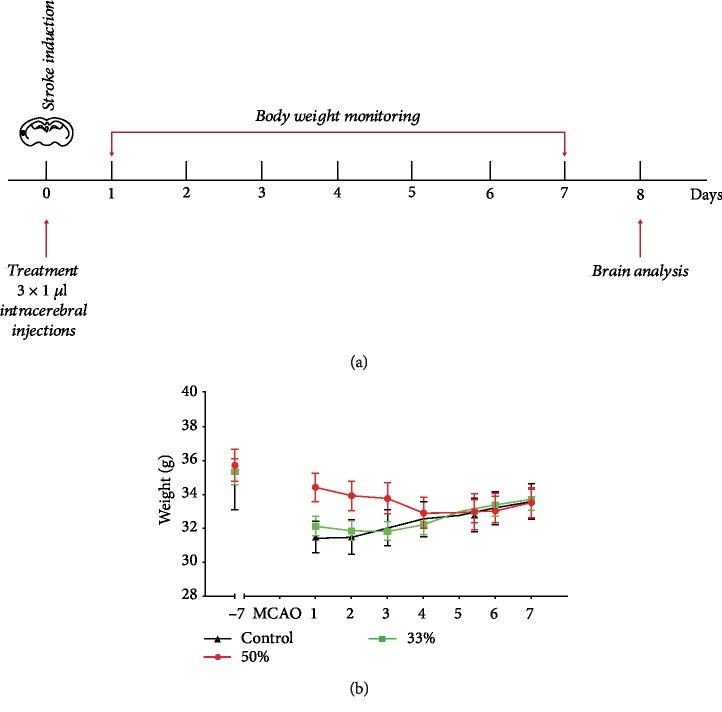
(a) Schematic overview of the experiment. (b) Body weight monitoring after stroke.

**Figure 2:**
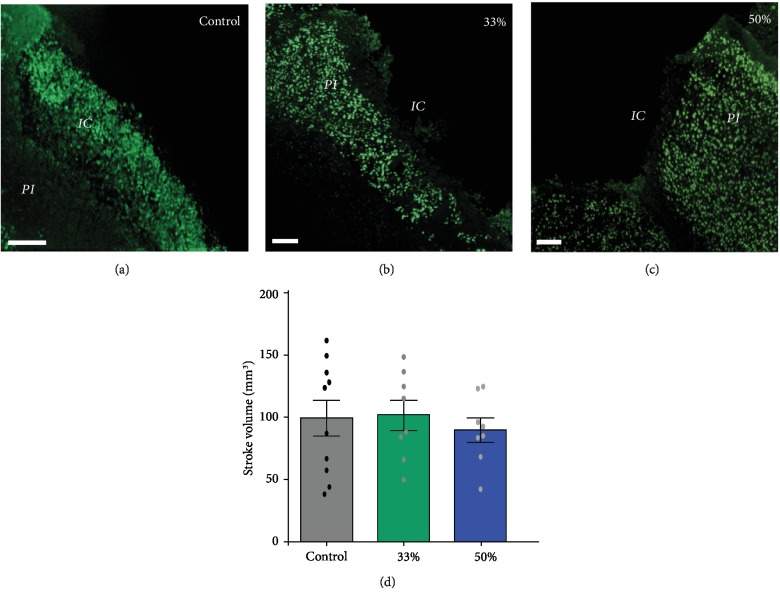
(a–c) Examples of the stroke size in the cortex, obtained by immunohistochemistry, revealed no differences in stroke volumes (d). *PI*: peri-infarct; *IC*: infarct core. Scale bar: (a–c) 100 *μ*m.

**Figure 3:**
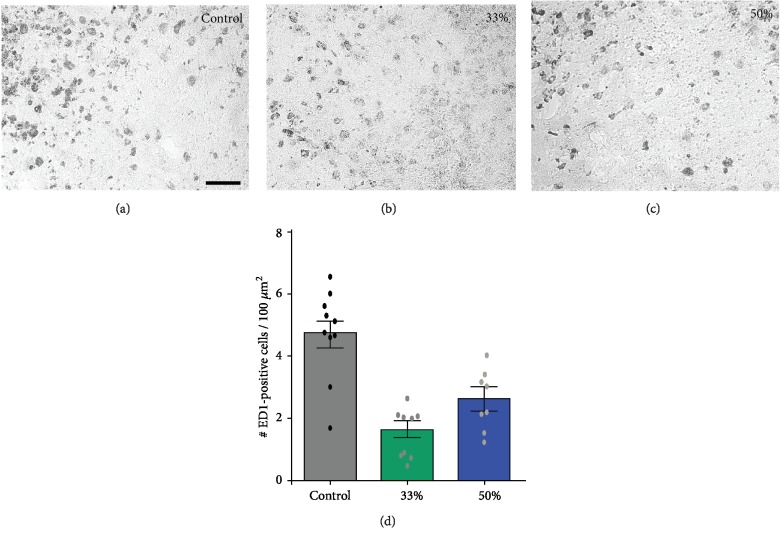
Quantification of histological images showed a decreased number of ED1-positive, phagocytic cells in the peri-infarcted area (F). The effect was more pronounced at the one third dose. Data is shown as mean ± SEM; ^∗∗∗^*p* < 0.001. Scale bar, 100 *μ*m.

Immediately after surgery, to prevent excessive weight loss, animals in all groups were fed soft pellets to facilitate nutrition. After a recovery period of 72 hours, all animals were permitted to eat ad libitum normal dry pellets. As expected, all groups lost body mass to some extent and started gaining weight after 4 days so that by day 7 there was no difference in body mass between the groups (Figure 1(b)). All animals in the third and control groups had a survival rate of 100%, while in the half-dose group, one animal died at the second day after stroke. However, the mortality rate was much higher in the group treated with full dose of SB290157 and we decided to discontinue treatment. The infarct volume at 7 days post MCA occlusion was similar in controls and treated animals, regardless of dose (Figures 2(a)–2(d)). However, when analyzing the cellular response of microglia, we found that animals treated with the C3R antagonist, SB290157, showed a 50% decrease in the number of phagocytic microglial cells at the half dose and a 75% decrease in the number of activated microglial cells at the one-third dose (Figure 3).

### 3.2. Preventing Inflammatory Morphological Transformation with C3aR Antagonist Injection

**Figure 4:**
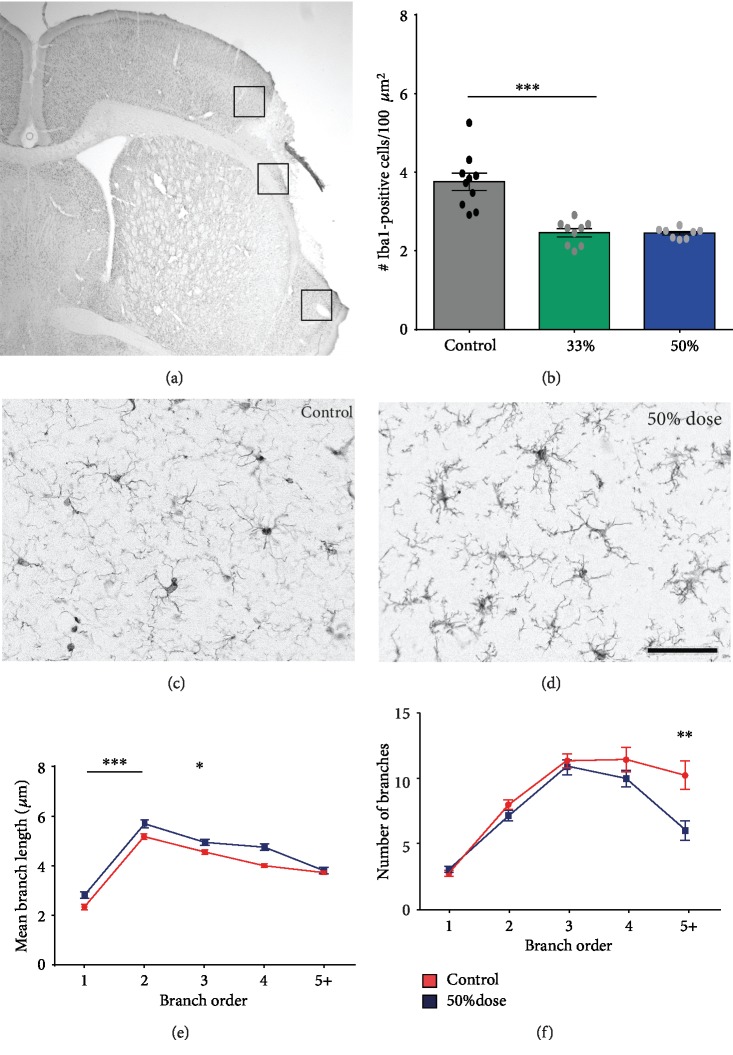
Local treatment with the C3aR antagonist revealed changes in Iba1-positive microglia morphology at two days after stroke. (a) Immunohistochemical images were taken from the penumbra in locations indicated by the rectangles. Quantification of Iba1-positive cells in the peri-infarcted area revealed a reduction in the number of cells expressing Iba1 by 40% (c–d) at the one-third dose. However, the dose-dependent effect was not pronounced (b). In the treated animals, Iba1-positive microglia exhibited longer first- and second-order branches as compared with controls (E; ^∗∗∗^p < 0.001), and a decreased number of terminal branches (f; ^∗∗^*p* < 0.01). The 3rd-order branches was less significant (^∗^*p* < 0.05). Values are given as mean ± SEM. Scale bar: 750 *μ*m (a) and 100 *μ*m (b, c).

The local administration of SB290157 attenuated the inflammatory changes in the morphology of microglia which were easily visible in the penumbra (Figures 4(a), 4(c), and 4(d)). Indeed, we found a reduction in the number of cells expressing Iba1 by 40% (Figures 4(b)–4(d)) at the one-third dose. However, the dose-dependent effect was not pronounced (Figure 4(b)).

By manually tracing microglia processes, we were able to identify that there was a decrease in the first, second, and third order of branching in the control group, showing that microglia in these animals are shortening their processes in preparation for an amoeboid transformation (Figure 4(e)). Also, there was a clear increase in the number of terminal processes in controls compared with treated animals, a clear sign of a decreased microglial activation in the treated group compared with the control (Figure 4(f)).

### 3.3. Intracortical Administration of SB290157 Impacts Both Microglial Migration and Phagocytosis

**Figure 5:**
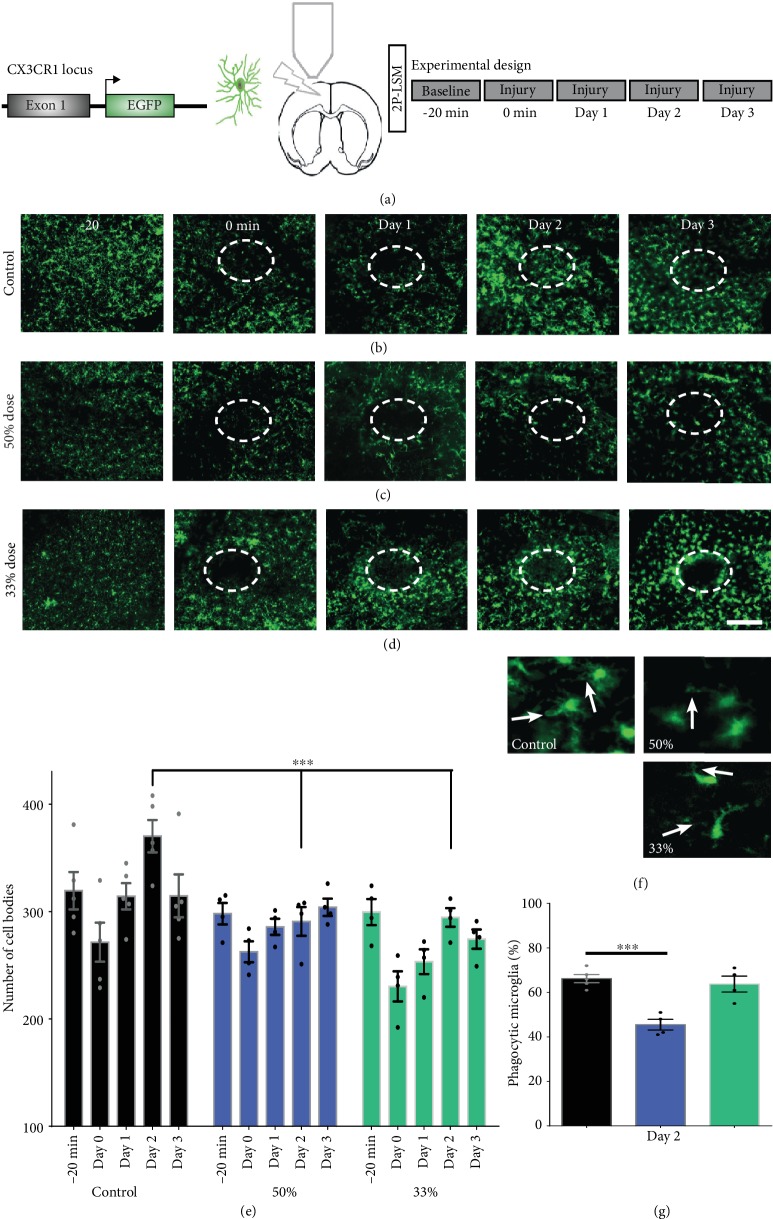
Representative two-photon microscopy time laps images of anesthetized mice. (a) Experiments were done on male CX_3_CR_1_^eGFP/-^ mice that underwent cranial window implantation over the right somatosensory cortex, (b–e) taken over a period of three days. During this period, microglial migration and morphology were recorded after a 1 mm deep stab wound injury done using a 30 G needle. Each experiment has a 20 min baseline (-20 min) for all group investigated. After the baseline, the injury is done and with the same needle the C3aR antagonist was locally administrated. The control group received only vehicle. (f) Quantification of the number of microglia over time, after the lesion, showed a decreased number of microglia around the lesion in the treated animals compared with the controls. (g) A dose-dependent response was observed when analyzing phagocytic microglia. Mean ± SEM; ^∗∗∗^*p* < 0.001. Scale bar: (b–e) 200 *μ*m and (g) 25 *μ*m.

Two-photon laser scanning microscopy has become a gold standard for *in vivo* cell behaviour analysis. The quantification of the number and morphology of microglia has been implicated in all neuropathologies, and the main conclusion is that both activation and inhibition are necessary for a healthy tissue recuperation [30]. By locally injecting the C3aR antagonist, we induced a small cortical lesion that was directly influenced by the diffusion of the antagonist. Although microglial migration did happen, at all doses of SB290157, after 48 h, there was a decrease in the number of microglial cells around the lesion (Figures 5(b)–5(f)). At the same time, we were able to see a dose-dependent decrease in the phagocytic capability of microglia, with less phagocytic phenotypes seen in the high-dose group compared with the lower ones or controls (Figures 5(g)).

## 4. Discussion

In this study, we investigated the effects of locally applied SB290157, an antagonist of the complement anaphylatoxin C3a receptor, on cortical microglia after a cortical lesion. We found that intracortical administration of SB290157 reduced the number of inflammatory microglial cells expressing ED1 and Iba1 antigens at the lesion site. We were also able to show, *in vivo*, that two days after a laser-induced cortical lesion, there were less activated microglia present around the injury site, displaying less high-order branches and an increase in the lower order ones, highly suggesting an attenuated phagocytic phenotype in treated animals as compared with controls.

It should be noted that a dose of 3 *μ*g SB290157 given intracortically to the poststroke mice led to a high mortality. A possible explanation is that at high doses, SB290157 can also function as a C3aR agonist in particular in cells having a high density of C3aRs [31–34]. Data on blood-brain barrier (BBB) penetration by SB290157 is not available. However, when given intracortically in the lesioned area, the penetration of blood-brain barrier (BBB) by SB290157 is not required because the BBB is already disrupted.

Although much is known about the role of immune cells and molecules, including cortical microglia and complement proteins, C1q [35] and C3 [36], during brain development and normal functioning in the adult, recent work has revealed that microglial cells are critical mediators of synaptic sculpting and reorganization via C3-dependent phagocytosis of synapses [37]. Following an acute injury such as stroke, the apoptotic neurons release a chemotactic signal such as fractalkine/CX3CL1 [12, 13] and microglia expressing the fractalkine receptor (CX3CR1), which promotes phagocytosis of apoptotic cells expressing CX3CL1 [13].

Activation and accumulation of C1q, C3c, and C4d around ischemic lesions make these signalling pathways attractive for stroke therapy [14]. Indeed, the depletion of certain complement components or inhibition of complement activation could reduce ischemic brain injury [17]. Clinical data also suggest that the levels of serum complement proteins and their activation products correlate with stroke severity [15, 16] and functional disability [17, 18]. Because microglia are among the first players of the CNS to an ischemic lesion [10], modulating how they will react during the acute phase might be a key to control remodeling processes during brain recovery and rehabilitation. Although C1q alone can promote both microglial clearance of misfolded proteins and apoptotic neurons, this process can be enhanced by C3 [19, 20].

## 5. Conclusion

In this study, we showed that the C3aR antagonist, SB290157 given intracortically, may be used in the future to limit neuroinflammation and, consequently, neuronal death after an ischemic lesion by modulating microglia transition to the phagocytic type and secondary phagocytosis.

## Data Availability

The data used to support the findings of this study are available from the corresponding author upon request.
